# Cardio-ankle vascular index for predicting cardiovascular morbimortality and determinants for its progression in the prospective advanced approach to arterial stiffness (TRIPLE-A-Stiffness) study

**DOI:** 10.1016/j.ebiom.2024.105107

**Published:** 2024-04-17

**Authors:** Magnus Bäck, Jirar Topouchian, Carlos Labat, Sylvie Gautier, Jacques Blacher, Marcin Cwynar, Alejandro de la Sierra, Denes Pall, Kevin Duarte, Francesco Fantin, Katalin Farkas, Luis Garcia-Ortiz, Zoya Hakobyan, Piotr Jankowski, Ana Jelakovic, Marina Kotsani, Alexandra Konradi, Oksana Mikhailova, Iveta Mintale, Oscar Plunde, Rafael Ramos, Anatoly Rogoza, Yuriy Sirenko, Nebojsa Tasic, Iurii Rudyk, Saule Urazalina, Peter Wohlfahrt, Parounak Zelveian, Roland Asmar, Athanase Benetos

**Affiliations:** a Department of Medicine Solna, Karolinska Institutet and Department of Cardiology Karolinska University Hospital, Stockholm, Sweden; b Inserm U1116, Nancy, France; c Université de Lorraine, CHRU Nancy, University Hospital of Nancy, France; d Paris-Descartes University, AP-HP, Diagnosis and Therapeutic Center, Hôtel Dieu, Paris, France; e Department of Internal Medicine and Gerontology, Jagiellonian University Medical College, Kraków, Poland; f Department of Internal Medicine, Hospital Mutua Terrassa, University of Barcelona, Terrassa, Spain; g Department of Medical Clinical Pharmacology, University of Debrecen, Hungary; h Department of Medicine, Section of Geriatric Medicine, University of Verona, Italy; i Cardiometabolic Centre, Dept. of Angiology, Szent Imre University Teaching Hospital, Budapest, Hungary; j Primary Care Research Unit of Salamanca (APISAL), Biomedical Research Institute of Salamanca (IBSAL), Department of Biomedical and Diagnostic Sciences, University of Salamanca, Salamanca, Spain; k Institute of Cardiology, Centre of Preventive Cardiology, Yerevan, Armenia; l Department of Internal Medicine and Geriatric Cardiology, Centre of Postgraduate Medical Education, Warsaw, Poland; m Department of Nephrology, Hypertension, Dialysis and Transplantation, University Hospital Centre, Zagreb, Croatia; n Almazov Federal Medical Research Centre, St-Petersburg, Russia; o FSBI “Chazov National Medical Research Centre of Cardiology” of the Ministery of Health of the Russian Federation, Moscow, Russia; p P. Stradins University Hospital, Cardiology Centre, Riga, Latvia; q Institut Universitari d’Investigació en Atenció Primària Jordi Gol, Department of Medical Sciences, University of Girona, Primary Care Services, Biomedical Research Institute, Institut Català de la Salut, Girona, Spain; r Institute of Cardiology, Kiev, Ukraine; s Medical Faculty, University of Belgrade and Cardiovascular Institute, Dedinje, Belgrade, Serbia; t Government Institution, L.T. Malaya Therapy Institute of the National Academy of Medical Sciences of Ukraine, Kharkov, Ukraine; u Scientific and Research Institute of Cardiology and Internal Diseases, Almaty, Kazakhstan; v Department of Preventive Cardiology, Institute for Clinical and Experimental Medicine, Prague, Czech Republic; w Foundation-Medical Research Institutes, Paris, France

**Keywords:** Arterial stiffness, Cardio-ankle vascular index, Cardiovascular morbimortality, Risk factor

## Abstract

**Background:**

The cardio-ankle vascular index (CAVI) measure of arterial stiffness is associated with prevalent cardiovascular risk factors, while its predictive value for cardiovascular events remains to be established. The aim was to determine associations of CAVI with cardiovascular morbimortality (primary outcome) and all-cause mortality (secondary outcome), and to establish the determinants of CAVI progression.

**Methods:**

TRIPLE-A-Stiffness, an international multicentre prospective longitudinal study, enrolled >2000 subjects ≥40 years old at 32 centres from 18 European countries. Of these, 1250 subjects (55% women) were followed for a median of 3.82 (2.81–4.69) years.

**Findings:**

Unadjusted cumulative incidence rates of outcomes according to CAVI stratification were higher in highest stratum (CAVI > 9). Cox regression with adjustment for age, sex, and cardiovascular risk factors revealed that CAVI was associated with increased cardiovascular morbimortality (HR 1.25 per 1 increase; 95% confidence interval, CI: 1.03–1.51) and all-cause mortality (HR 1.37 per 1 increase; 95% CI: 1.10–1.70) risk in subjects ≥60 years. In ROC analyses, CAVI optimal threshold was 9.25 (c-index 0.598; 0.542–0.654) and 8.30 (c-index 0.565; 0.512–0.618) in subjects ≥ or <60 years, respectively, to predict increased CV morbimortality. Finally, age, mean arterial blood pressure, anti-diabetic and lipid-lowering treatment were independent predictors of yearly CAVI progression adjusted for baseline CAVI.

**Interpretation:**

The present study identified additional value for CAVI to predict outcomes after adjustment for CV risk factors, in particular for subjects ≥60 years. CAVI progression may represent a modifiable risk factor by treatments.

**Funding:**

International Society of Vascular Health (ISVH) and 10.13039/100023010Fukuda Denshi, Japan.

**Research in context**

**Evidence before this study**

Currently used methods to evaluate arterial stiffness predict cardiovascular outcomes, but are not recommended as part of the cardiovascular risk assessment. Cardio-ankle vascular index (CAVI) is a novel measure of arterial stiffness.

**Added value of this study**

This study measured arterial stiffness and its progression with time using the Cardio Ankle Vascular Index in over 1000 individuals across Europe.

✓ Stiffer arteries increased the risk of cardiovascular diseases.
✓ Arterial stiffness measurement by Cardio Ankle Vascular Index may improve the predictability and treatment for reducing cardiovascular disease.

**Implications of all the available evidence**

The present study extends the established associations of CAVI with cardiovascular risk by showing that CAVI progression was affected by risk factors as well as being potentially modifiable by treatments. The use of CAVI in cardiovascular risk prediction and guidance merit further studies.

## Introduction

Cardio-ankle vascular index (CAVI) is a measure of arterial stiffness,^1^ which is associated with cardiovascular (CV) risk factors in cross-sectional studies. A vast majority of studies were performed in Asian populations but have subsequently been replicated in non-Asian populations.^2^^,^^3^

Advanced Approach to Arterial Stiffness (TRIPLE-A-Stiffness) is the largest CAVI study in Europe, enrolling more than two thousand subjects ≥40 years old at 32 centres from 18 European countries.^2^ The cross-sectional analysis of TRIPLE-A-Stiffness reported a strong correlation with age for CAVI and that age-adjusted CAVI was associated with components of the metabolic syndrome. A significant positive association was observed for CAVI with glycemia and hypertension, whereas the waist circumference was inversely associated with CAVI.^2^

Subjects with established atherosclerotic CV disease are at high risk of CV events. Comparative studies have shown a remarkable difference of CAVI in subjects with and without established atherosclerosis.^3^^,^^4^ This may in part reflect higher incidence of co-morbidities, such as diabetes, hypertension, and hyperlipidaemia, although prospective studies are needed to determine the predictive value of CAVI for incident and recurrent CV events.

A meta-analysis of the existing, mainly small and including specific risk populations, prospective studies reported a modest association between CAVI and incident CV risk.^5^ The latter report highlighted the need for studies assessing CAVI as a predictor of CV disease in the general population and non-Asian countries.

The aim of this study was to establish the association of CAVI with CV morbimortality (primary outcome) and all-cause mortality (secondary outcome), and to establish the determinants of CAVI progression. To this end, the TRIPLE-A-Stiffness participants were followed prospectively for monitoring of outcomes and serial CAVI measures.

## Methods

### Study design

TRIPLE-A-Stiffness is an international multicentre prospective longitudinal study with three scheduled visits at baseline and after 2 and 5 years of follow-up. Subjects aged 40 years and older were recruited at 32 centres in 18 countries. All subjects were followed in outpatient clinics for prevention check-up and/or monitoring of CV risk factors. Non-inclusion criteria were factors potentially impairing the quality and reliability of arterial stiffness measurements. The exclusion criteria were: (a) known significant peripheral arterial disease, (b) ankle–brachial index bilaterally less than 0.95, (c) limb amputation; (d) history of vascular surgery of the carotid artery, femoral artery or aorta; (e) BMI more than 40 kg/m2; (f) atrial fibrillation and/or other major arrhythmia; and (g) pregnancy. The study is registered at www.clinicaltrials.gov with the ID number: NCT02318628.

### Prospective study population

Participants enrolled in TRIPLE-A-Stiffness,^2^ for whom follow-up was available were included in this study. The flow chart is shown in Supplementary Fig. S1. The cross-sectional analysis of the baseline examinations has been previously reported.^2^ The target size for the cross-sectional analysis was at least 2000 subjects, and reached an inclusion of 2325 subjects. Of these, 54% were included in the prospective analysis based on available data on events during follow-up from n = 985 subjects, follow-up with serial CAVI measures for n = 921 subjects, and both outcome measures for n = 656 subjects (Supplementary Table S1).

### Clinical evaluation at baseline

Investigators collected data from physical examination, disease history and treatments. Sex was self-reported by the participants. Blood pressure measurements were performed after 5–10 min rest according to the ESH/ESC guidelines for the management of arterial hypertension^6^ using validated equipment that meets certification criteria. Two or more readings were averaged. If the first two readings differed by more than 15 mmHg, additional readings were obtained and averaged. The average values of the BP measurements were reported. Blood samples for bioassays or reporting of the results of the laboratory examination performed at ±12 weeks of the baseline visit date.

### Cardio-ankle vascular index

CAVI was measured using the VaSera system (Fukuda Denshi Co, Japan) as previously described.^2^ A microphone for phonocardiography was placed on the sternum, and BP cuffs were placed at the four limbs. CAVI was automatically calculated by the VaSera CAVI using the heart-ankle PWV to estimate the β-stiffness according to the following equation^7^:

$$\text{CAVI}=a\left\{\frac{2\rho }{\text{PP}}\;x\;\left(\ln\frac{\text{SBP}}{\text{DBP}}\right)\;x\;{\text{PWV}}^{2}\right\}+b$$

where a and b are adjustable constants, ρ blood density (1.05), PP pulse pressure, SBP and DBP systolic and diastolic blood pressure, PWV pulse wave velocity from the origin of the aorta to the tibial artery. According to the automated quality control in the VaSera, results were not reported if acquisition was of insufficient quality. Only measurements deemed valid by the device were included. The mean value of the left and right CAVI was used. CAVI values to stratify participants in three different groups were: CAVI < 8; 8–9, and >9.

### Follow-up

All subjects examined at baseline and attending the 2 and/or 5-year follow-up visits were included. At follow-up information was collected on CV morbimortality. Information on cause of mortality was collected from patient records and/or information from the person contacted when a diseased subject was invited for attending a follow-up visit. The investigator noted if CV morbimortality had occurred, and classified the event as “cardiac”, “cerebral”, or “vascular”. The cardiac morbimortality included all cardiac diagnoses and procedures (*e.g.* myocardial infarction, vascular interventions, heart failure etc). The cerebral cause was defined as cerebrovascular disease (stroke, TIA), whereas other CV events and cause of mortality were recorded as vascular. The follow-up data was collected by the participating centres without event adjudication by the coordinator. CAVI measures were performed at the follow-up visit. If atrial fibrillation was prevalent at the follow-up visit, CAVI measure was not performed. A manual quality control was performed by an experience investigator (JT). The exclusion for quality were: Age < 39.5, duplicated visit, missing date of birth, missing CAVI results at either baseline or follow-up visit, and bilateral ABI <0.95. If ABI was <0.95 unilaterally, the contralateral CAVI was used. The change in CAVI was calculated as the last available CAVI measure minus the baseline value and CAVI progression was obtained by dividing the change in CAVI by the time in years between the two examinations.

### Statistics

Continuous variables are presented as mean ± SD or median (IQR) for skewed distribution, and discrete variables are presented as frequency and percentage. The 2-tailed significance level was set at P < 0.05. Pairwise comparisons were carried out using the Mann–Whitney and χ2 tests as appropriate. The assumptions for applying χ2 tests were checked and found to be non-violated for all comparisons. Unadjusted incidence rates (IRs) and cumulative IRs during the follow-up time for CV morbimortality and all-cause mortality were displayed as Kaplan–Meier graphs. Follow-up started at the date of baseline visit and subjects were censored at the date of event (CV event for CV morbimortality analysis, death for all-cause mortality analysis) or at the date of latest news. Origin and start times for survival analysis were the same. Subgroup analysis was performed after stratification by age of either ≥60 or <60 years based on predefined age-limits and a target sample size of 500 subjects per subgroup. Cox proportional hazards models were used to assess unadjusted and adjusted associations for incident CV morbimortality and all-cause mortality. Covariates, chosen based on clinical judgment and literature, included age (as a continuous variable), sex, smoking, BMI, mean arterial blood pressure (MAP), dyslipidemia, diabetes, prevalent CV disease, chronic kidney disease (CKD), anti-hypertensive, anti-diabetic, and lipid lowering treatments. Cox models were adjusted for the above-mentioned covariates except medications. For each endpoint, an optimal cut-off of CAVI was determined by maximizing Harrell’s c-index in univariable Cox model. Pearson’s correlation was used to study the relationship between annual CAVI progression, baseline CAVI and age. To identify predictors of yearly CAVI progression adjusted for baseline CAVI, covariates with a P < 0.10 in an univariable linear regression model (adjusted for baseline CAVI) were included in the multivariable model. The statistical analyses were performed using the NCSS 9 statistical software package (Kaysville, Utah, USA).

### Ethics

The protocol with reference ANSM ID RCB 2014-A01754-43 was approved by Comité de protection des personnes (CPP) Ile de France 1 and put forth for ethical approval in each participating country. Informed written consent was obtained for all included subjects.

### Role of funders

The sources of funding had no access to the study data and no role in the design, implementation or reporting.

## Results

### Subject characteristics

The baseline characteristics of the n = 1250 subjects included in the prospective study (Supplementary Fig. S1) shown in Table 1 were consisted with a high CV risk population. In comparison with the n = 1074 subjects examined at baseline^2^ but not followed up in the prospective study sample exhibited a higher proportion of smoking, prevalent comorbidities, and pharmacological/non-pharmacological treatments (Table 1). The prospective study sample had significantly higher proportions of lipid-lowering and hypertension treatments, with concomitant better lipid levels and blood pressure compared with the subjects with missing follow-up. In contrast, the arterial stiffness measures were not significantly different between the subjects with and without follow-up (Table 1). The participation at baseline and follow-up for each participating centre is shown in Supplementary Table S2.

**Table 1:** Baseline characteristics of the initially included subjects and in the 1250 subjects with follow-up for at least one of the primary, secondary, and CAVI are included in the prospective cohort.

Subjects with follow-up	No	Yes	P
n	1075	1250	
Age (years)—mean (SD)	59 (11)	60 (11)	0.093
Female sex—no. (%)	586 (55%)	633 (51%)	0.062
Current smoker—no. (%)	245 (23%)	183/1249 (15%)	<0.0001
Body Mass Index (kg/m^2^)—mean (SD)	29.30 (4.83)	29.30 (4.57)	0.82
Waist circumference (cm)—mean (SD)	100 (14)	101 (12)	0.39
Metabolic syndrome—no. (%)	743/1016 (73%)	890/1174 (75%)	0.15
SBP (mmHg)—mean (SD)	142 (18)	139 (18)	<0.0001
DBP (mmHg)—mean (SD)	86 (11)	84 (11)	<0.0001
MAP (mmHg)—mean (SD)	104 (12)	102 (12)	<0.0001
Medical history—no. (%)			
CVD	360 (33%)	409 (33%)	0.69
Hypertension	834/1051 (79%)	1011/1241 (81%)	0.20
Dyslipidemia	764/1048 (73%)	871/1231 (71%)	0.26
Diabetes	252/1054 (24%)	286/1240 (23%)	0.63
CKD	39/1020 (4%)	72/1230 (6%)	0.027
Family history of CVD	372/944 (39%)	439/1086 (40%)	0.64
Medications—no. (%)			
Anti-hypertensive treatment	742/1070 (69%)	976/1248 (78%)	<0.0001
Lipid-lowering	454/1071 (42%)	639/1248 (51%)	<0.0001
Anti-diabetic treatment	206/1070 (19%)	230/1248 (18%)	0.61
Laboratory measures			
Total cholesterol (mmol/l)—median (IQR)	5.31 (4.64–6.30) (n = 1009)	5.17 (4.40–6.00) (n = 1167)	<0.0001
LDL (mmol/l)—median (IQR)	3.20 (2.42–4.04) (n = 985)	2.92 (2.19–3.80) (n = 1110)	<0.0001
HDL (mmol/l)—median (IQR)	1.32 (1.08–1.60) (n = 852)	1.35 (1.11–1.66) (n = 1102)	0.039
TG (mmol/l)—median (IQR)	1.40 (1.02–1.97) (n = 982)	1.37 (1.02–1.90) (n = 1144)	0.22
Fasting glucose (mmol/l)—mean (SD)	6.01 (1.88) (n = 978)	5.95 (1.80) (n = 1134)	0.92
Arterial stiffness measures			
CAVI (dimensionless)—mean (SD)	8.26 (1.33)	8.35 (1.35)	0.11
Mortality and CV morbimortality			
CV morbimortality—no. (%)	–	129/985 (13%)	
CV morbimortality follow-up (years)—median (IQR)	–	3.78 (2.64–4.67) (n = 985)	
Total mortality—no. (%)	–	54/985 (5%)	
Total mortality follow-up (years)—median (IQR)	–	3.82 (2.80–4.69) (n = 985)	

The number of subjects (n) in each group is indicated in the top row of and n is specified for each variable with missing values. Results are reported as either mean (SD) or median (IQR) for continuous variables and n (%) for categorical variables. Statistical differences were evaluated using Mann–Whitney and χ2 tests as appropriate.

### Predictive value of baseline CAVI for outcomes

**Fig. 1:**
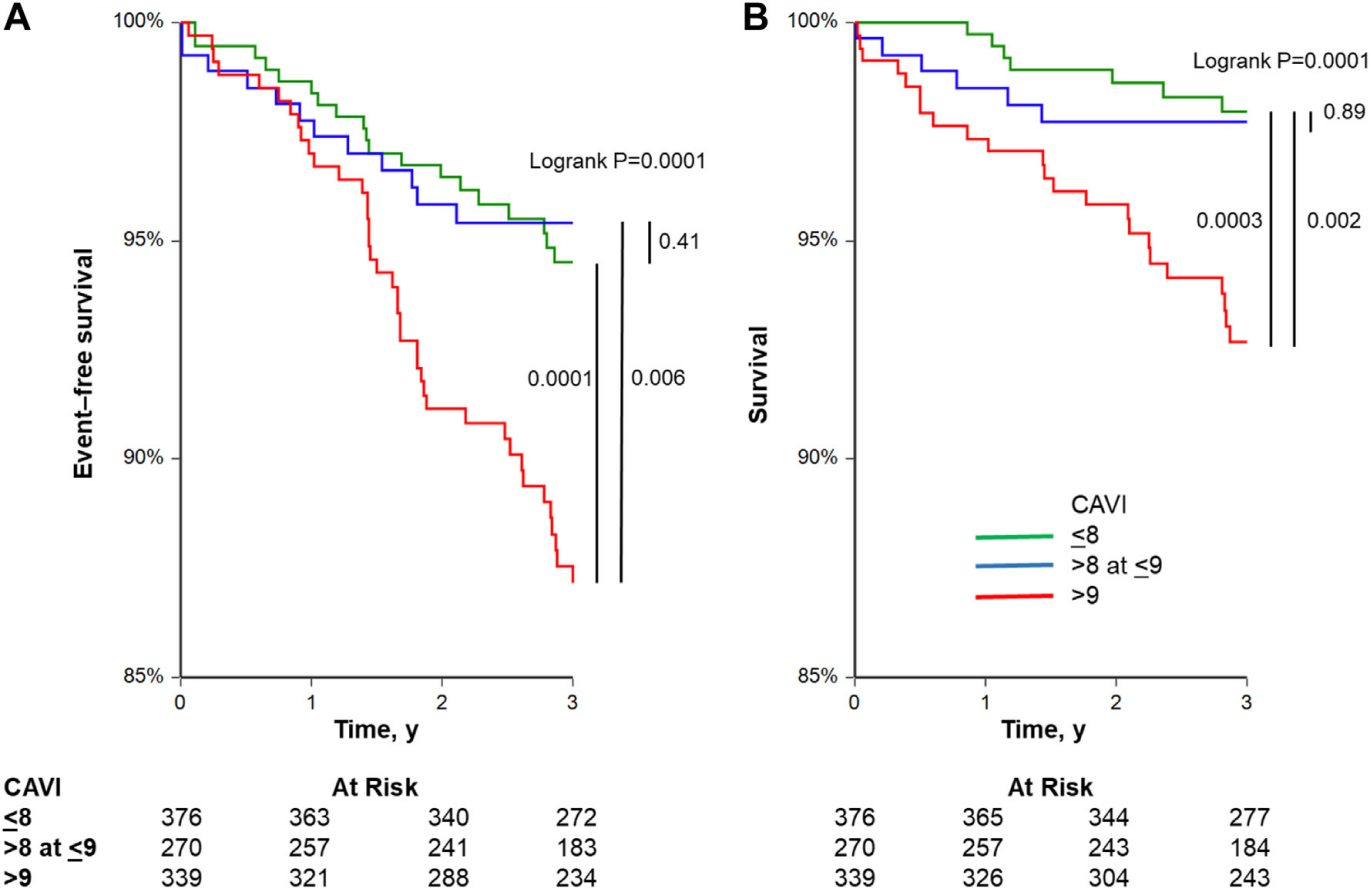
Kaplan Meier plots for cumulative (a) cardiovascular morbimortality and (b) all-cause mortality according to the CAVI stratum at baseline. Red: CAVI > 9, blue: CAVI 8–9, and green: CAVI < 8.

During a median follow-up time of 3.82 (IQR 2.81–4.69) years, 129 subjects experienced an event in the primary CV morbimortality outcome, and 54 subjects for the secondary outcome of all-cause mortality (Table 1). The components of the CV morbimortality are shown in Supplementary Table S3. Cardiac cause represented more than half of the outcomes, followed by cerebral and vascular events. In thirteen cases, the primary CV morbimortality outcome was not distinguishable, for which the outcome was marked as other (Supplementary Table S3). The unadjusted cumulative incidence rates according to CAVI at baseline were higher in highest stratum (CAVI >9) compared with either the lowest (CAVI < 8) or second (CAVI 8–9) strata, whereas no significant differences were observed between the first and second strata (Fig. 1A). A similar pattern was observed for the secondary outcome all-cause mortality (Fig. 1B).

**Fig. 2:**
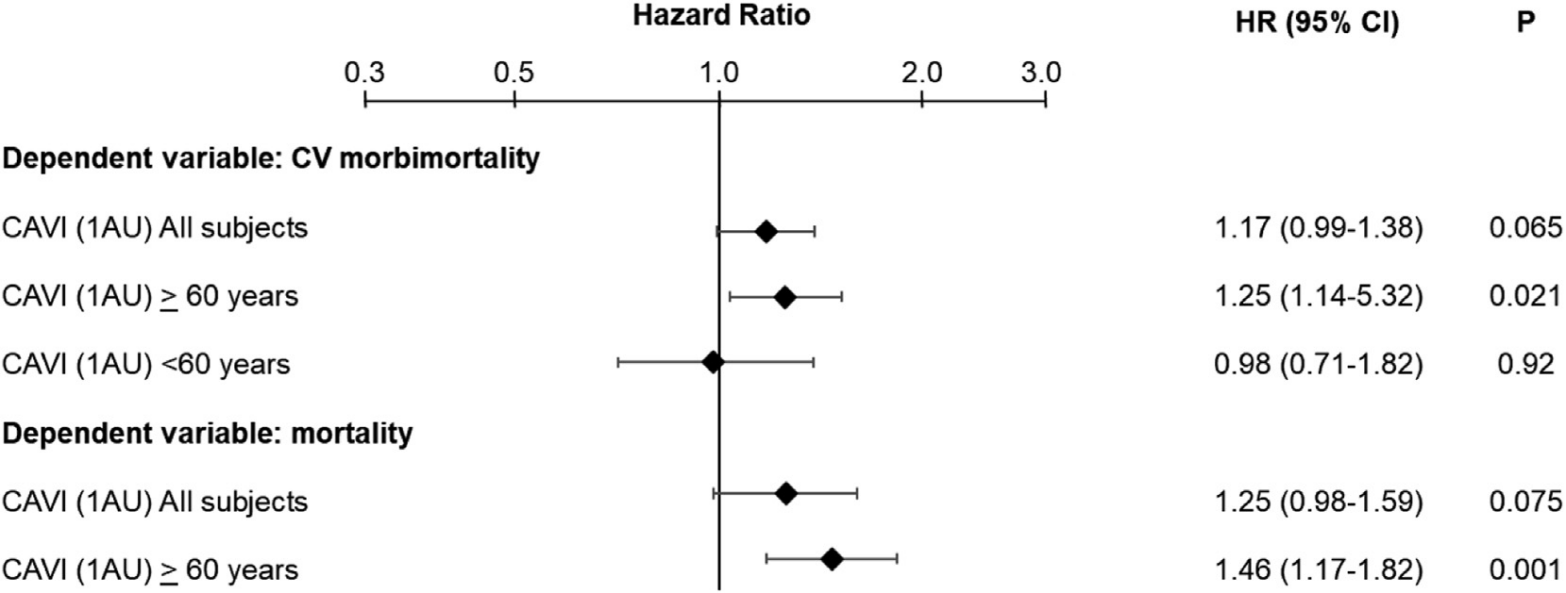
Hazard ratios for cardiovascular (CV) morbimortality and all-cause mortality per 1 arbitrary unit (AU) CAVI increase from a fully adjusted (Model 2) Cox analysis in the whole population and in age-stratified subgroups.

A Cox regression with CAVI as a continuous variable after age- and sex adjustment (Model 1) revealed a significant association after age stratification. In subject 60 years or older at baseline, every 1-point increment in CAVI was associated with an increased risk of the primary composite outcome of CV morbimortality (HR, 1.27, 95% CI, 1.06–1.52). whereas no significant association was detected in subjects below 60 years of age (Table 2). In a Cox regression adjusted for sex, age, and confounding variables for baseline CAVI: smoking, BMI, MAP, dyslipidaemia, diabetes, prevalent CV disease, and prevalent CKD (Model 2), CAVI was associated with an increased risk in 60 years or older subjects at a HR of 1.25 (95% CI, 1.03–1.51) (Fig. 2), with significant associations for age, prevalent CVD, and smoking (Supplementary Fig. S2). In an age- and sex-adjusted Cox regression adjusted for anti-hypertensive, anti-diabetic, and lipid lowering treatments (Model 3), CAVI remained a risk predictor in all (HR 1.20, 95% CI 1.02–1.40) and subjects ≥60 years (HR 1.30, 95% CI 1.09–1.55).

**Table 2:** Age- and sex-adjusted Cox regressions for the HR for every 1-point increment in CAVI (CAVI 1U) and the individual co-variate for the primary (cardiovascular morbidity-mortality) and secondary (all-cause mortality) outcomes.

	All	P	≥60 years	P	<60 years	P
Cardiovascular morbimortality						
CAVI 1U	1.17 (1.00–1.38)	0.051	1.27 (1.06–1.52)	0.009	0.98 (0.72–1.34)	0.89
Age (10 y)	1.57 (1.29–1.92)	<0.0001	1.76 (1.32–2.35)	0.0001	1.24 (0.69–2.22)	0.47
Female sex	0.80 (0.56–1.14)	0.22	1.07 (0.70–1.65)	0.76	0.43 (0.21–0.86)	0.017
All-cause mortality						
CAVI 1U	1.21 (0.96–1.52)	0.10	1.37 (1.10–1.71)	0.005	N/A	
Age (10 y)	2.78 (2.03–3.81)	<0.0001	3.19 (2.14–4.75)	<0.0001	N/A	
Female sex	0.42 (0.24–0.74)	0.003	0.49 (0.26–0.90)	0.021	N/A

The secondary outcome of all-cause mortality was observed in 54 subjects, of which 45 subjects were ≥60 years of age. Given the few mortality cases in the group <60 years, analyses for all-cause mortality were performed only for the subgroup of subjects ≥60 years (Table 2). Results for Model 2 (Fig. 2) revealed similar associations in the ≥60 years group for 1-point increment in CAVI with all-cause mortality as observed for CV morbimortality, remaining significant after adjustments for treatments (HR 1.37, 95% CI 1.10–1.70).

### CAVI thresholds for outcomes

In ROC analyses, the CAVI limits for prediction of outcome were different in the age groups, with an optimal threshold of CAVI 9.25 in subjects ≥60 years for CV morbimortality and CAVI 9.95 for all-cause mortality (Table 3). Cox-analyses with CAVI dichotomized according to optimal thresholds of revealed a prediction in crude analyses as well as after adjustment for age and sex (Table 4). In the fully adjusted analysis (Model 2), CAVI superior to the threshold was associated with a 1.5 and 1.6-fold increased risk for CV morbimortality in all and ≥60 years subjects, respectively (Table 4). Likewise, the CAVI threshold of 9.95 significantly predicted all-cause mortality in subjects ≥60 years (Table 4). In contrast, the CAVI threshold of 8.3 in subjects <60 years was not significantly associated with CV morbimortality (Table 4).

**Table 3:** Optimal threshold for CAVI for each outcome for all and the age subgroups (<60 years, ≥60 years).

	Optimal thresholds^a^	C-index (CI 95%) for CAVI > s
Cardiovascular morbimortality		
All	9.25	0.606 (0.559–0.653)
<60 years	8.30	0.565 (0.512–0.618)
≥60 years	9.25	0.598 (0.542–0.654)
All-cause mortality		
All	9.60	0.667 (0.596–0.738)
≥60 years	9.95	0.659 (0.581–0.737)

a The optimal threshold was determined by maximizing Harrell’s c-index.

**Table 4:** Association of CAVI dichotomized (optimal threshold) with outcomes (CV morbimortality, total mortality) in Cox models.

	Univariable model		Model adjusted for age and sex		Model adjusted for age and sex + other clinical factors^a^	
	HR	P	HR	P	HR	P
Cardiovascular morbimortality						
All CAVI > 9.25	2.53 (1.78–3.58)	<0.0001	1.49 (0.99–2.23)	0.054	1.54 (1.02–2.34)	0.042
≥60 years CAVI > 9.25	2.17 (1.42–3.33)	0.0003	1.62 (1.02–2.56)	0.039	1.62 (1.00–2.62)	0.049
<60 years CAVI >8.30	0.80 (0.38–1.68)	0.56	0.68 (0.32–1.46)	0.33	0.69 (0.31–1.54)	0.37
All-cause mortality						
All	5.03 (2.95–8.59)	<0.0001	1.57 (0.85–2.90)	0.15	1.36 (0.70–2.64)	0.36
≥60 years	4.37 (2.43–7.87)	<0.0001	2.06 (1.08–3.92)	0.028	2.29 (1.12–4.69)	0.023

a Other clinical factors included BMI, CV disease, smoker, MAP, diabetes, dyslipidemia and renal failure.

### CAVI progression

During follow-up, the median CAVI progression was 0.07 (−0.10 to +0.25)/year. Yearly CAVI progression was inversely correlated with the baseline CAVI level (R^2^ = 0.1035; Supplementary Fig. S3A) but not correlated with age (R^2^ = 0.0014; P = 0.26; Supplementary Fig. S3B). Nevertheless, yearly CAVI progression was positively correlated with age within the two lower baseline CAVI strata (<8 and 8–9), with a trend for a positive correlation in the highest strata with baseline CAVI > 9 (Supplementary Fig. S3C).

To determine the univariate associations with confounding factors independently of baseline CAVI, CAVI yearly progression was adjusted for baseline CAVI. In this analysis, age and mean blood pressure were associated with adjusted CAVI progression, and a trend was observed for lipid-lowering treatment, in the univariate analyses (Supplementary Table S4).

Multivariable regression to determine predictors of CAVI progression adjusted for baseline-CAVI revealed age, mean arterial blood pressure as well as anti-diabetic and lipid-lowering treatment as independent predictors of CAVI progression (Supplementary Table S5). In a stratified analysis, the crude and age-adjusted baseline CAVI was higher in the presence compared with the absence of CVD (Supplementary Table S6). In contrast, the CAVI progression adjusted for baseline CAVI was similar between the groups (Supplementary Table S6).

## Discussion

The findings of the present study point to CAVI strata predicting of CV morbimortality and all-cause mortality in a population ≥40 years with increased prevalence of CV risk factors. The predictive value of CAVI was strongest in subjects ≥60 years, for who the CAVI thresholds established in the present study remained significant risk predictors even after adjustment of CV risk factors. Finally, the CAVI progression was monitored and identified age and MAP as well as lipid-lowering and anti-diabetic treatments as independent determinants of CAVI progression underlying the implications of CAVI as a measure of modifiable CV risk.

The inclusion criteria in Triple A-Stiffness were wide to be representative of the general population. More than half of the population participated in the prospective part of the study. The follow-up cohort had higher proportions of treatments and better hypertension and dyslipidaemia management. It should however be noted that only one third had prevalent CVD with lower baseline CAVI, making it a suitable population for initiation of prevention in case CAVI would lead to reclassification.

The stratification according to the CAVI in the whole population revealed significantly increased incidence of the primary endpoint CV morbimortality and the secondary outcome of all-cause mortality in the highest compared with the two lower strata. The CAVI absolute values were substantially lower compared with one of the first prospective CAVI study, which was a single centre study including 400 subjects with prevalent hypertension, diabetes, dyslipidaemia, or a history of CV disease. The baseline CAVI was substantially higher compared with the present study, and stratification by CAVI < 9; 9–10, and >10 showed an approximately doubled risk for CVD event incidence in the highest compared with the lowest CAVI groups after adjustment for the unequal distribution of CV risk factors between the groups.^8^ A meta-analysis of in total 3 prospective trials comparing the highest and lowest CAVI based on >10 vs <9, ≥9.9 vs <8, or ≥ vs <9 yielded an overall non-significant HR of 1.34.^5^ The prospective CAVI-J study in an Asian population showed significant increased HR for CV events and all-cause mortality from a CAVI of 9.5 (vs lowest quintile at CAVI ≤ 7.55).^9^ The differences in the crude analysis of CAVI strata in the present study hence extends previous observation of higher CAVI risk for CV morbimortality and all-cause mortality to lower risk populations with lower baseline CAVI and by showing its applicability in non-Asian populations.

Cardiac causes represented more than half of the CV morbimortality in the present study. For the CAVI-J trial secondary outpoints, heart failure was among the highest HR pointing to CAVI predicting a more than 3-fold increased risk for heart failure hospitalization. This is further supported by a small study of 154 subjects with and without heart failure associated a baseline CAVI > 9.56 with a an approximately 1.4-fold increase in death or HF-related hospital admission during a mean of 2.56 years follow-up.^10^ Increased CAVI is in addition associated with subclinical cardiac affection detected on echocardiography, in terms of increased left ventricular mass and decreased systolic function.^11^

Cox regressions adjusting for age and sex, CV risk factors, and pharmacological treatments identified a significant association of CAVI with the primary outcome measure of CV morbimortality. Findings are consistent with a meta-analysis including 4 cohorts of subjects with either metabolic disorders^12^, ^13^, ^14^ or coronary artery disease,^15^ in which the overall risk increase was 20% per 1 CAVI arbitrary unit (AU) increment.^5^ The most recent and largest multicentre prospective CAVI-J trial of subjects with CV risk factors^16^ included 2938 subjects in Japan and showed per 1-point CAVI-increment a 1.4-fold increased risk of composite CV events in terms of CV death, myocardial infarction, and stroke after a median follow-up of 4.9 years.^9^ The strongest and significant association was observed in subjects ≥60 years, which raises the notion of the additional predictive value of CAVI in the risk assessment of older subjects.

The optimal CAVI threshold for prediction of CV morbimortality was also substantially higher in older compared with younger subjects. Importantly, CAVI superior to 9.25 was an independent predictor of CV morbimortality after adjustment for CV risk factors. These findings support the additional value of CAVI evaluation for CV risk evaluation in European populations, which have previously evoked in Asian populations.^9^ The present study in addition extends the previous findings of a significant risk prediction by CAVI for both CV morbimortality and all-cause mortality in a non-Asian general population with lower CV risk and lower baseline CAVI measures. It should however be noted that all-cause mortality analysis was limited to subjects ≥60 years and that age dependent CAVI thresholds may be applicable for CV morbimortality risks evaluation.

The serial evaluation of CAVI in the present study revealed a yearly progression of 0.07 CAVI AU. Importantly, CAVI at baseline was the main determinant of CAVI progression. Nevertheless, within CAVI strata, age was a significant predictor of CAVI progression. This indicates that a low CAVI should be re-evaluated for a continuous monitoring of reaching the threshold for being considered as an additional CV risk factor. When adjusting for baseline CAVI the present study identified age and MAP as well as lipid-lowering and anti-diabetic treatments as independent predictors of CAVI progression. These findings provide a suggestion that CAVI progression maybe a modifiable risk factor. Statin-treatment is associated with a significant reduction in arterial stiffness measured as aortic augmentation index.^17^ In addition, serial CAVI assessment for prediction of future CV events has previously been shown in patients with coronary artery disease.^15^ In contrast, no significant CAVI progression was reported at 6 months in a study of patients with significant carotid stenosis undergoing carotid artery stenting.^4^

### Caveats and limitations

The present study supports CAVI as a predictor of CV morbimortality and identifies predictors of CAVI progression. There are however some limitations, which should be acknowledged. First, the CV morbimortality was only specified as cardiac, cerebrovascular, or vascular, and 10% had a non-distinguishable primary cause. Further studies are hence needed to provide a more granular outcome analysis. Second, the younger part of the population experienced fewer CV events and low mortality, which indicates that larger studies are needed to determine the predictive value of CAVI in younger more healthier subjects. Third, the general applicability of the findings in this population with a high proportion of CV risk factors remains to be established. Fourth, some CV events occurred before the second CAVI measures, precluding an assessment of the prognostic value of CAVI progression for CV morbidity in the present study. Fifth, the multicentre design of the study cannot exclude variations between centres although previous studies have documented high reproducibility for CAVI measures.^18^ In addition, the study was preceded by a certification procedure to ensure that all centres were performing CAVI measurements homogeneously according to a standardized method with high quality and reproducibility. Sixth, the lack of validation cohort precluded the validation of the predictive performance of having a CAVI above the optimal threshold. Finally, methodological limitations of the study design and analysis, including that no causality can be definitely attributed to the associations and that unmeasured residual confounding, measurement errors and variable exposure time to confounders,^19^ as well as the possible selection bias in hazard ratios cannot be excluded.

### Conclusion

In conclusion, CAVI is evolving as a useful tool in CV risk determination, with additional value after adjustment for CV risk factors, in particular for subjects ≥60 years. In addition, the present study extends the established associations of CAVI with prevalent CV risk factors, to CAVI progression being affected by CV risk factors as well as being potentially modifiable by treatments. The use of CAVI in cardiovascular risk prediction and guidance merit further studies.

## Contributors

JT, RA, AB contributed to the conception or design of the work. All authors (MB, JT, CL, SG, JB, MC, AS, DP, KD, FF, KF, LGA, Zh, PJ, AJ, MK, AK, OM, IM, OP, RR, AR, YS, NT, IR, SU, PW, PZ, RA, AB) contributed to the acquisition of data for the work. MB, CL, JT, RA, AB performed the analysis, verification, and interpretation of the data. MB, JT, RA, AB drafted the manuscript. All authors (MB, JT, CL, SG, JB, MC, AS, DP, KD, FF, KF, LGA, Zh, PJ, AJ, MK, AK, OM, IM, OP, RR, AR, YS, NT, IR, SU, PW, PZ, RA, AB) have reviewed the manuscript.

## Data sharing statement

Individual participant data that underlie the results reported will be shared, after deidentification, with researchers who provide a methodologically sound proposal.

*Time Frame:* Beginning 9 months following article publication and finishing 36 months following article publication.

*Access Criteria:* Investigators interested in data should contact the corresponding author.

## Declaration of interests

AdlS reports support from Sanofi and Viatris. JB reports support from AstraZeneca, Bayer, Elkendi, Hikma, Leurquin, Omron, Organon, Sanofi, and Vivactis. AK reports honoraria for lecturing from Servier, KRKA, and Novartis, and travel support from Servier. PW reports support from Ministry of Health of the Czech Republic, grant nr. NV 19-09-00125, National Institute for Research of Metabolic and Cardiovascular Diseases (Programme EXCELES, Project No. LX22NPO5104)–Funded by the European Union–Next Generation EU, Servier, and ProMED. The remaining authors have nothing to disclose.

## Appendix BSupplementary data

Supplementary Figs. S1–S3 and Tables S1–S6

Study Details ClinicalTrials
