# Supplementary material for: Cardio-ankle vascular index for predicting cardiovascular morbimortality and determinants for its progression in the prospective advanced approach to arterial stiffness (TRIPLE-A-Stiffness) study
Source: eBioMedicine. 2024 Apr 17;103:105107. doi: 10.1016/j.ebiom.2024.105107 (PMC11121166; doi:10.1016/j.ebiom.2024.105107)

<u>**Supplement**</u>

Supplementary Table 1 Baseline subgroup characteristics

Follow-up for CV morbimortality and all-cause mortality	***Yes***	***Yes***	
Follow-up with serial CAVI measures	***Yes***		***Yes***
n	*656*	*985*	*921*
Age (years) – mean (SD)	*60 (10)*	*61 (11)*	*59 (10)*
Female sex – no. (%)	*334 (51%)*	*501 (51%)*	*466 (51%)*
Current smoker – no. (%)	*95/655 (15%)*	*136/984 (14%)*	*142/920 (15%)*
Body Mass Index (kg/m²) – mean (SD)	*29.27 (4.56)*	*29.32 (4.55)*	*29.26 (4.58)*
Waist circumference (cm)) – mean (SD)	*101 (13)*	*102 (12)*	*100 (13)*
Metabolic syndrome – no. (%)	*470/602 (78%)*	*721/918 (79%)*	*639/858 (74%)*
SBP (mmHg)) – mean (SD)	*139 (17)*	*139 (18)*	*139 (17)*
DBP (mmHg)) – mean (SD)	*84 (11)*	*84 (11)*	*85 (11)*
MAP (mmHg)) – mean (SD)	*103 (12)*	*102 (12)*	*103 (12)*
***Medical history** – no. (%)*			
CVD	*207 (32%)*	*319 (32%)*	*297 (32%)*
Hypertension	*543/649 (84%)*	*806/976 (83%)*	*748/914 (82%)*
Dyslipidemia	*453/644 (70%)*	*680/966 (70%)*	*644/909 (71%)*
Diabetes	*132/649 (20%)*	*251/977 (26%)*	*167/912 (18%)*
CKD	*34/643 (5%)*	*65/965 (7%)*	*41/908 (5%)*
Family history of CVD	*255/655 (43%)*	*343/874 (39%)*	*351/808 (43%)*
***Medications** – no. (%)*			
Anti-hypertensive treatment	*530/655 (81%)*	*794/983 (81%)*	*712/920 (77%)*
Lipid-lowering	*345/655 (53%)*	*519/983 (53%)*	*464/920 (50%)*
Anti-diabetic treatment	*114/655 (17%)*	*199/983 (20%)*	*145/920 (16%)*
Laboratory measures			
Total cholesterol (mmol/L) – median (IQR)	*5.13 (4.40 – 5.91) (n=591)*	*5.10 (4.30 – 5.90) (n=903)*	*5.20 (4.40 – 6.03) (n=855)*
LDL (mmol/L) – median (IQR)	*2.82 (2.15 – 3.78) (n=571)*	*2.82 (2.10 – 3.65) (n=851)*	*2.98 (2.20 – 3.90) (n=830)*
HDL (mmol/L) – median (IQR)	*1.37 (1.14 – 1.71) (n=562)*	*1.35 (1.14 – 1.69) (n=854)*	*1.35 (1.11 – 1.65) (n=810)*
TG (mmol/L) – median (IQR)	*1.38 (1.03 – 1.97) (n=579)*	*1.38 (1.05 – 1.93) (n=881)*	*1.38 (1.00 – 1.92) (n=842)*
Fasting glucose (mmol/L) – mean (SD)	*5.91 (1.78) (n=577)*	*6.02 (1.86) (n=873)*	*5.86 (1.72) (n=836)*
***Arterial stiffness measures** –*			

CAVI (dimensionless) – mean (SD)	*8.34 (1.22)*	*8.45 (133)*	*8.23 (1.27)*
Follow-up Time *(years)*			
CV morbimortality – mean (SD)	*3.71 (1.45)*	*3.78 (1.57)*	-
All-cause mortality – mean (SD)	*3.79 (1.41)*	*3.87 (1.55)*	-
CAVI – mean (SD)	*3.16 (0.91)*	-	*3.16 (1.02)*
Follow-up Results			
CV morbimortality – no. (%)	*59 (9%)*	*129 (13%)*	-
All-cause mortality – no. (%)	*6 (1%)*	*54 (5%)*	-
CAVI progression (U/year) – mean (SD)	*0.039 (0.405)*	-	*0.063 (0.396)*

Supplementary Table 1: Baseline characteristics in subgroups followed for subsequent events (n=985), serial CAVI measures (n=921), and both outcome measures available (n=656). Results are reported as either mean (SD) or median (IQR) for continuous variables and n (%) for categorical variables. The number of subjects (n) in each group is indicated in the top row of and n is specified for each variable with missing values.

Supplementary Table 2 Geographical distribution of study participants

Country Center	Baseline visit	Follow Up	Serial measures of CAVI	Cardiovascular morbi-mortality and all cause mortality
	N	N and (%) of Baseline vist		
Armenia	**251**	**158 (63%)**	**101 (40%)**	**158 (63%)**
ARM1	*173*	*94 (54%)*	*57 (33%)*	*94 (54%)*
ARM2	*78*	*64 (82%)*	*44 (56%)*	*64 (82%)*
Belarus	**82**	**26 (32%)**	**26 (32%)**	**0 (0%)**
BEL1	*82*	*26 (32%)*	*26 (32%)*	*0 (0%)*
Croatia	**98**	**84 (86%)**	**56 (57%)**	**84 (86%)**
CRO1	*98*	*84 (86%)*	*56 (57%)*	*84 (86%)*
Czech Republic	**27**	**18 (67%)**	**17 (63%)**	**18 (67%)**
CZE1	*27*	*18 (67%)*	*17 (63%)*	*18 (67%)*
France	**111**	**55 (50%)**	**33 (30%)**	**55 (50%)**
FRA1	*103*	*55 (53%)*	*33 (32%)*	*55 (53%)*
FRA2	*8*	*0 (0%)*	*0 (0%)*	*0 (0%)*
Greece	**104**	**0 (0%)**	**0 (0%)**	**0 (0%)**
GRE1	*54*	*0 (0%)*	*0 (0%)*	*0 (0%)*
GRE2	*50*	*0 (0%)*	*0 (0%)*	*0 (0%)*
Hungary	**174**	**72 (41%)**	**57 (33%)**	**72 (41%)**
HUN1	*93*	*32 (34%)*	*27 (29%)*	*32 (34%)*
HUN2	*81*	*40 (49%)*	*30 (37%)*	*40 (49%)*
Italy	**133**	**82 (62%)**	**56 (42%)**	**82 (62%)**
ITA1	*116*	*82 (71%)*	*56 (48%)*	*82 (71%)*
ITA2	*17*	*0 (0%)*	*0 (0%)*	*0 (0%)*
Kazakhstan	**100**	**75 (75%)**	**75 (75%)**	**0 (0%)**
KAZ1	*100*	*75 (75%)*	*75 (75%)*	*0 (0%)*
Latvia	**82**	**57 (70%)**	**34 (41%)**	**55 (67%)**
LAT1	*64*	*55 (86%)*	*32 (50%)*	*55 (86%)*
LAT2	*18*	*2 (11%)*	*2 (11%)*	*0 (0%)*
Lebanon	**66**	**0 (0%)**	**0 (0%)**	**0 (0%)**
LEB1	*66*	*0 (0%)*	*0 (0%)*	*0 (0%)*
Lithuania	**118**	**0 (0%)**	**0 (0%)**	**0 (0%)**
LIT1	*118*	*0 (0%)*	*0 (0%)*	*0 (0%)*
Poland	**167**	**111 (66%)**	**105 (63%)**	**70 (42%)**
POL1	*69*	*41 (59%)*	*41 (59%)*	*0 (0%)*
POL2	*98*	*70 (71%)*	*64 (65%)*	*70 (71%)*
Russia	**232**	**59 (25%)**	**17 (7%)**	**59 (25%)**

RUS1	*70*	*59 (84%)*	*17 (24%)*	*59 (84%)*
RUS2	*48*	*0 (0%)*	*0 (0%)*	*0 (0%)*
RUS3	*63*	*39 (62%)*	*39 (62%)*	*0 (0%)*
RUS4	*90*	*0 (0%)*	*0 (0%)*	*0 (0%)*
Serbia	**102**	**59 (58%)**	**55 (54%)**	**59 (58%)**
SER1	*102*	*59 (58%)*	*55 (54%)*	*59 (58%)*
Spain	**148**	**103 (70%)**	**99 (67%)**	**83 (56%)**
SPA1	*29*	*20 (69%)*	*20 (69%)*	*0 (0%)*
SPA2	*119*	*83 (70%)*	*79 (66%)*	*83 (70%)*
SPA3	*101*	*97 (96%)*	*0 (0%)*	*97 (96%)*
Sweden	**39**	**30 (77%)**	**28 (72%)**	**30 (77%)**
SWE1	*39*	*30 (77%)*	*28 (72%)*	*30 (77%)*
Ukraine	**151**	**125 (83%)**	**123 (81%)**	**63 (42%)**
UKR1	*76*	*62 (82%)*	*62 (82%)*	*0 (0%)*
UKR2	*75*	*63 (84%)*	*61 (81%)*	*63 (84%)*
Total	**2325**	**1250**	**921**	**985**

Supplementary Table 3

	N	*%*
Cardiovascular morbidity-mortality	***129***	***100%***
Cardiac	*68*	*52.7%*
Cerebrovascular	*26*	*20.2%*
Vascular	*22*	*17.0%*
Other	*13*	*10.1%*

Supplementary Table 3: Distribution of the cardiovascular morbidity-mortality categories defined as the outcome measures in the follow-up.

Supplementary Table 4

	Beta (CI 95%)	*P*
Age (10 y)	*0.095 (0.066 to 0.124)*	*<0.0001*
Female sex	*0.005 (-0.053 to 0.043)*	*0.85*
Current smoker	*-0.055 (-0.123 to 0.012)*	*0.11*
Body Mass Index (kg/m^2)	*0.001 (-0.005 to 0.006)*	*0.82*
MAP (10 mmHg)	*0.025 (0.010 to 0.045)*	*0.017*
Medical history		
CVD	*0.001 (-0.052 to 0.054)*	*0.97*
Dyslipidemia	*0.014 (-0.041 to 0.068)*	*0.62*
Diabetes	*0.054 (-0.010 to 0.119)*	*0.10*
CKD	*0.060 (-0.058 to 0.178)*	*0.32*
Medications		
Anti-hypertensive treatment	*-0.008 (-0.067 to 0.050)*	*0.78*
Lipid-lowering	*-0.049 (-0.099 to 0.000)*	*0.052*
Anti-diabetic treatment	*0.071 (0.003 to 0.139)*	*0.041*

Supplementary Table 4. Multivariable associations for yearly CAVI progression adjusted for baseline CAVI.

Supplementary Table 5

	Beta (CI 95%)	*Decrease in R^2 if variable was removed (%)*	*P*
CAVI 1U	*-0.154 (-0.178 to -0.131)*	*14.7%*	*<0.0001*
Age (10 y)	*0.109 (0.080 to 0.139)*	*4.8%*	*<0.0001*
MAP (10 mmHg)	*0.035 (0.015 to 0.055)*	*1.1%*	*0.0006*
Lipid-lowering	*0.073 (0.006 to 0.139)*	*1.0%*	*0.001*
Anti-diabetic treatment	*-0.082 (-0.131 to -0.033)*	*0.4%*	*0.033*
	**Overall model :**	*Adjusted R^2 = 0.1621*	

Supplementary Table 5. Multiple regression for independent predictors of CAVI progression adjusted for baseline CAVI

Supplementary Table 6

	No CVD (n=841)	*CVD (n=409)*	Unadjusted		Age-adjusted		Initial value adjusted	
			MD/RD (CI 95%)	p-value	MD (CI 95%)	p-value	MD (CI 95%)	p-value
Age (y) – mean (SD)	58 ± 11	63 ± 10	-4.3 (-5.5 to -3.0)	<0.0001	-	-	-	-
Women – n (%)	463 (55%)	170 (42%)	13.5 (7.7 to 19.3)	<0.0001	-	-	-	-
Baseline CAVI (U) – mean (SD)	8.20 (1.36)	8.65 (1.29)	-0.45 (-0.61 to -0.30)	<0.0001	-0.132 (-0.260 to -0.004)	0.042		
CAVI progression (U/year) – mean (SD)	0.079 (0.36)	0.029 (0.46)	0.049 (-0.005 to 0.104)	0.076	0.045 (-0.011 to 0.101)	0.12	-0.001 (-0.054 to 0.052)	0.97

MD: mean difference; RD: risk difference.

Supplementary Table 6: CAVI at baseline and progression according to the absence or presence of a history of CVD.

Supplementary Fig 1: Study flow-chart

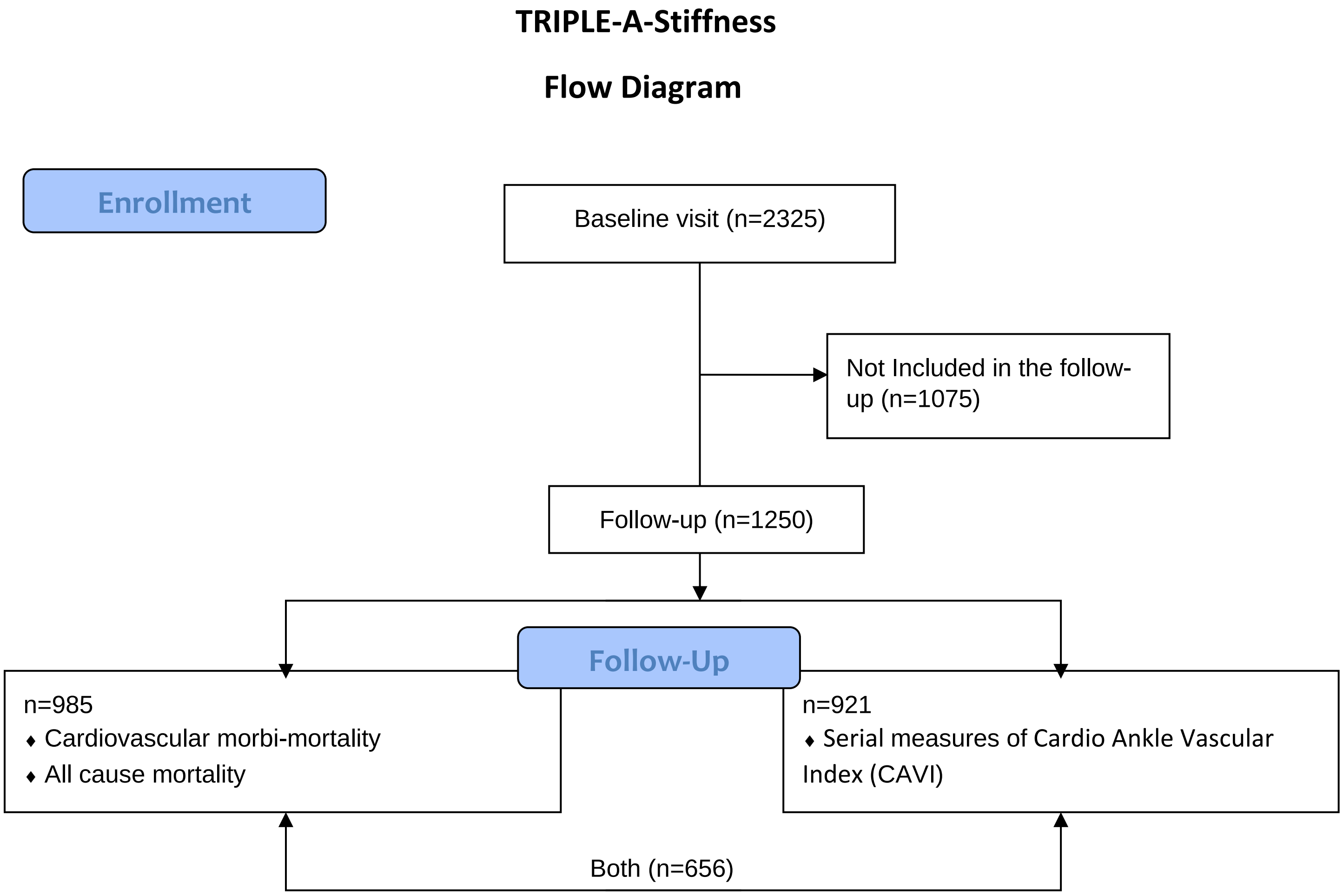

Supplementary Figure 2: Hazard ratio (HR) for covariates in Model 2 in the Cox analysis for subjects ≥ 60 years

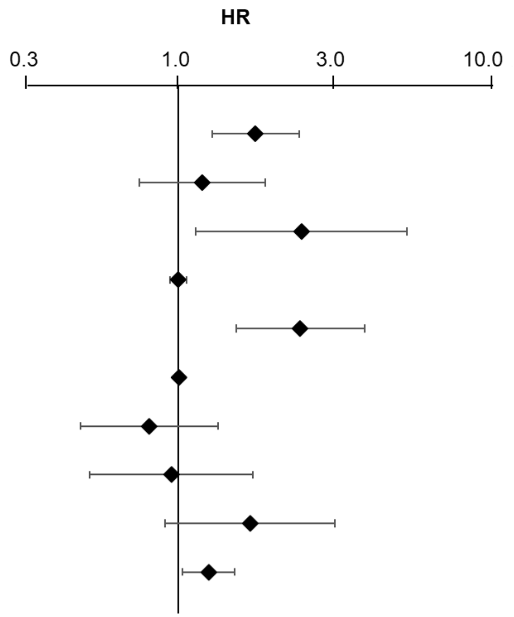

Independent variable	Hazard Ratio (95% CI)	P
Age (10 y)	1.76 (1.28-2.43)	0.0005
Women, yes	1.19 (0.75-1.89)	0.45
Smoker, yes	2.46 (1.14-5.32)	0.020
BMI (1 kg/m2)	1.00 (0.94-1.06)	0.97
CVD (yes)	2.44 (1.53-3.91)	0.0002
MBP (1mmHg)	1.00 (0.99-1.02)	0.63
Diabetes (yes)	0.81 (0.49-1.34)	0.41
Dyslipidemia (yes)	0.95 (0.52-1.73)	0.87
Renal Failure (yes)	1.69 (0.91-3.15)	0.097
CAVI (1AU)	1.25 (1.03-1.51)	0.022

Supplementary Figure 3: Relationships of yearly CAVI progression with age and baseline CAVI.

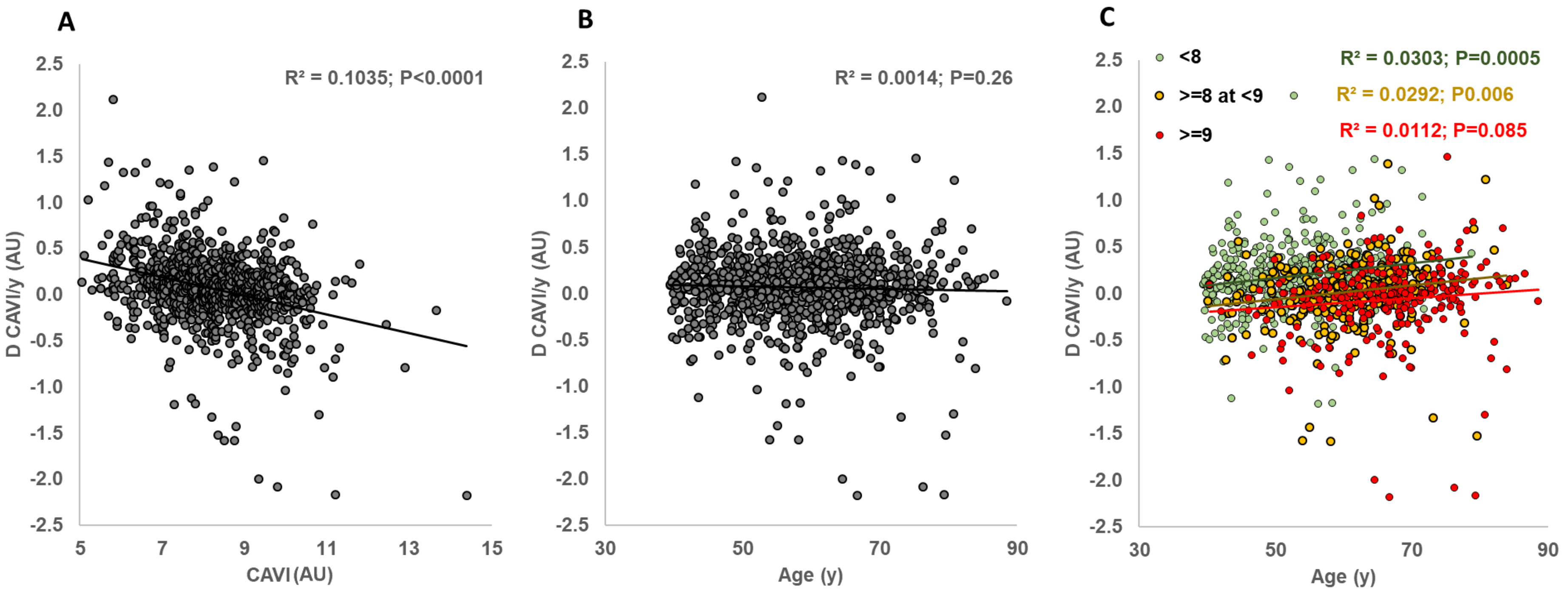

Supplement: Supplementary Figs. S1–S3 and Tables S1–S6 [file mmc1.pdf]
