# Supplementary material for: Cardio-ankle vascular index for predicting cardiovascular morbimortality and determinants for its progression in the prospective advanced approach to arterial stiffness (TRIPLE-A-Stiffness) study
Source: eBioMedicine. 2024 Apr 17;103:105107. doi: 10.1016/j.ebiom.2024.105107 (PMC11121166; doi:10.1016/j.ebiom.2024.105107)
Supplement: Study Details ClinicalTrials [file mmc2.pdf]

The U.S. government does not review or approve the safety and science of all studies listed on this website.

Read our full disclaimer (https://clinicaltrials.gov/about-site/disclaimer) for details.

COMPLETED

Advanced Approach to Arterial Stiffness (AAA)

ClinicalTrials.gov ID NCT02318628

Sponsor International Society for Vascular Health

Information provided by International Society for Vascular Health (Responsible Party)

Last Update Posted 2018-02-05

Study Details Tab

Study Overview

Brief Summary

The aim of the 3A Study is to assess the role of MetS on the arterial mechanics and vascular health in different age groups using the Cardio Ankle Vascular Index (CAVI) of the Vasera system and the "classic" carotid-femoral Pulse Wave Velocity (PWV).

Detailed Description

Feedback

Hypothesis The aim of thie 3A study is to assess the role of MetS on the arterial mechanics and vascular health in different age groups using the Cardio Ankle Vascular Index (CAVI) of the Vasera system and the "classic" carotid-femoral Pulse Wave Velocity (PWV).

STUDY OBJECTIVES

1. Objectives Primary Objective: Assess the influence of MetS on CAVI values (primary study endpoint) in 3 different age groups (40-54, 55-69, 70-85 years) of a European population.
2. Secondary Objectives
    - Establish the CAVI values in a European population according to the clusters of (MetS) components i.e. cardiovascular risk factors: abdominal obesity, high triglycerides, low HDL cholesterol, elevated blood pressure (BP), systolic or diastolic, and elevated fasting glucose (and diabetes), as well as tobacco smoking.
    - Assess the correlation between CAVI and the carotid-femoral PWV in the full population and in each age group; and compare the impact of MetS and its components on both the CAVI and PWV
    - To establish, the relationship between cardiovascular risk factors and the evolution of arterial stiffness as evaluated by CAVI and carotid-femoral PWV over a 2-year follow-up period.
    - To assess changes and predictive value of CAVI on organ damages and CV events during the follow-up period.
    - To assess 24h ABPM and its correlations and variations with the CAVI and PWV in patients with MetS and/or its different components.

This is a multinational European prospective longitudinal study.

Official Title

Advanced Approach to Arterial Stiffness (AAA Study - Triple A Study)

Conditions

Arterial Stiffness

Intervention / Treatment

- Device: Cardio Ankle Vascular Index (CAVI) of the Vasera system VS1500N

Other Study ID Numbers

- ISVH3A2013

Study Start

2014-12-01

Primary Completion (Actual)

2016-06-01

Study Completion (Actual)

2016-12-01

Enrollment (Actual)

2000

Study Type

Observational

Resource links provided by the National Library of Medicine

Other U.S. FDA Resources (https://classic.clinicaltrials.gov/ct2/info/fdalinks)

Contacts and Locations

This section provides the contact details for those conducting the study, and information on where this study is being conducted.

France

Paris, France, 75016
ISVH

Participation Criteria

Researchers look for people who fit a certain description, called eligibility criteria. Some examples of these criteria are a person's general health condition or prior treatments.

For general information about clinical research, read Learn About Studies (https://clinicaltrials.gov/study-basics/learn-about-studies).

Eligibility Criteria

Description

Inclusion Criteria:

- All patients aged 40-85 with life expectancy over 2 years will be included. Patients will be stratified according to their age (3 groups: 40-54, 55-69, 70-85 years)) and the presence or not of MetS.

Exclusion Criteria:

- Factors that may impair the quality of the CAVI and /or the PWV measurement or make PWV recording unreliable: known significant peripheral vascular disease with proximal artery stenosis, ankle brachial index < 0.9, or limb amputation; history of vascular surgery at the level of the carotid artery, femoral artery or aorta; body mass index > 40 kg/m^2; atrial fibrillation and/or other major arrhythmia.

Study Population

All patients aged 40-85 with life expectancy over 2 years will be included. Patients will be stratified according to their age (3 groups: 40-54, 55-69, 70-85 years)) and the presence or not of MetS.

Ages Eligible for Study

40 Years to 85 Years (Adult, Older Adult)

Sexes Eligible for Study

All

Accepts Healthy Volunteers

No

Sampling Method

Non-Probability Sample

Study Plan

This section provides details of the study plan, including how the study is designed and what the study is measuring.

How is the study designed?

What is the study measuring?

Primary Outcome Measures

Outcome Measure	Measure Description	Time Frame
Cardio-Ankle-Vascular Index	CAVI will be measured using the Vasera device (Fukuda Denshi co, Japan). This device is easy to use and calculates automatically the CAVI.<br><br>CAVI measurements will be performed according to the manufacturer recommendations Measurements will be performed after five to ten minute - period of rest in order to obtain a steady hemodynamic state and to limit measurements variability.<br><br>The details of the CAVI measurements are shown in a specific document, C.F. appendix<br><br>Ankle Brachial Index (ABI):<br><br>The Ankle Brachial Index (ABI) will be measured using the Vasera device (Fukuda Denshi co, Japan). This device will calculate the ABI automatically.<br><br>ABI measurements will be performed according to the manufacturer recommendations Measurements will be performed after five to ten minute - period of rest in order to obtain a steady hemodynamic state and to limit measurements variability.	2 years

Secondary Outcome Measures

Outcome Measure	Measure Description	Time Frame
Pulse Wave Velocity	Measurement of PWV will be performed using a validated automatic device (Complior®, Sphygmocor®, and PulsePen®). The method of	2 years

	"simultaneous" recordings of the pulse waves from 2 different sites (carotid and femoral arteries) will be preferred. Because of the use of several devices, normalisation of the measurement values will be performed according to the European Experts recommendations.	
Blood pressure Measurements	Blood pressure will be measured in clinic according to the European Society of Hypertension (ESH) guidelines. BP should be measured in a standardized fashion using equipment that meets certification criteria.	2 YEARS

Collaborators and Investigators

This is where you will find people and organizations involved with this study.

Sponsor

International Society for Vascular Health

Collaborators

No information provided

Investigators

- Study Director: JIRAR TOPOUCHIAN, ISVH

Publications

The person responsible for entering information about the study voluntarily provides these publications. These may be about anything related to the study.

General Publications

No publications available

* Find Publications about Study Results and related Pubmed Publications in the “Results” section of the study record.

Study Record Dates

These dates track the progress of study record and summary results submissions to ClinicalTrials.gov. Study records and reported results are reviewed by the National Library of Medicine (NLM) to make sure they meet specific quality control standards before being posted on the public website.

Study Registration Dates

First Submitted
2014-12-12

First Submitted that Met QC Criteria
2014-12-16

First Posted (Estimated)
2014-12-17

Study Record Updates

Last Update Submitted that met QC Criteria
2018-02-02

Last Update Posted
2018-02-05

Last Verified
2018-02

More Information

Terms related to this study

Keywords Provided by International Society for Vascular Health

Cardio ankle vascular index
CAVI

pulse wave velocity

PWV

Non invasive arterial structure assessment

Study Documents

No study documents available
